# Machine Learning Analytics of Resting-State Functional Connectivity Predicts Survival Outcomes of Glioblastoma Multiforme Patients

**DOI:** 10.3389/fneur.2021.642241

**Published:** 2021-02-22

**Authors:** Bidhan Lamichhane, Andy G. S. Daniel, John J. Lee, Daniel S. Marcus, Joshua S. Shimony, Eric C. Leuthardt

**Affiliations:** ^1^Department of Neurological Surgery, Washington University School of Medicine, St. Louis, MO, United States; ^2^Department of Biomedical Engineering, Washington University in St. Louis, St. Louis, MO, United States; ^3^Mallinckrodt Institute of Radiology, Washington University School of Medicine, St. Louis, MO, United States; ^4^Department of Neuroscience, Washington University School of Medicine, St. Louis, MO, United States; ^5^Department of Mechanical Engineering and Materials Science, Washington University in St. Louis, St. Louis, MO, United States; ^6^Center for Innovation in Neuroscience and Technology, Washington University School of Medicine, St. Louis, MO, United States; ^7^Brain Laser Center, Washington University School of Medicine, St. Louis, MO, United States

**Keywords:** brain tumor, resting state functional connectivity, biomarker, overall survival, short and long-term survival, classification, support vector machine

## Abstract

Glioblastoma multiforme (GBM) is the most frequently occurring brain malignancy. Due to its poor prognosis with currently available treatments, there is a pressing need for easily accessible, non-invasive techniques to help inform pre-treatment planning, patient counseling, and improve outcomes. In this study we determined the feasibility of resting-state functional connectivity (rsFC) to classify GBM patients into short-term and long-term survival groups with respect to reported median survival (14.6 months). We used a support vector machine with rsFC between regions of interest as predictive features. We employed a novel hybrid feature selection method whereby features were first filtered using correlations between rsFC and OS, and then using the established method of recursive feature elimination (RFE) to select the optimal feature subset. Leave-one-subject-out cross-validation evaluated the performance of models. Classification between short- and long-term survival accuracy was 71.9%. Sensitivity and specificity were 77.1 and 65.5%, respectively. The area under the receiver operating characteristic curve was 0.752 (95% CI, 0.62–0.88). These findings suggest that highly specific features of rsFC may predict GBM survival. Taken together, the findings of this study support that resting-state fMRI and machine learning analytics could enable a radiomic biomarker for GBM, augmenting care and planning for individual patients.

## Introduction

Glioblastoma multiforme (GBM) is the most frequently occurring brain malignancy. The median survival time is very poor, ranging from 12 to 14.6 months following diagnosis and after receiving the therapeutic standard of care (1, 2). Only 3–5% of GBM patients survive longer than 3 years after diagnosis (3). Facing such abbreviated lifespans, decisions of care balancing aggressiveness of treatment with impacts on quality of life, are critical to patients. As a result, the identification of novel prognostic biomarkers may have substantial and meaningful impact for individual patients making decisions for their terminal care.

Currently, a tissue diagnosis is required for definitive histopathologic confirmation and optimizing the next steps of care. Factors currently known to be associated with survival include age, Karnofsky performance status (KPS) (4), O6-methylguanine–DNA methyltransferase promoter (MGMT) hypermethylation (5), and mutations in isocitrate dehydrogenase (IDH) 1 or 2 (6, 7). Furthermore, gene expression–based molecular classification of GBM (8), epidermal growth factor receptor amplification (EGFR) (9) and CpG island methylator phenotype status have emerged as additional potential predictors of treatment response and outcome (10). Although such molecular information has improved the clinical assessment of GBM and has been used to better inform clinical trials (5–8, 10, 11), there remains unmet clinical need for accessible, non-invasively acquired biomarkers that predict clinical prognosis and response to therapy for individual patients *prior to surgical intervention and biopsy*.

There are numerous efforts in imaging radiomics to map image features to molecular data. As an example, investigators have correlated quantitative computed tomography (CT) image features to gene expression data of non-small cell lung cancer to predict survival (12, 13). Similarly in GBM, prior work has demonstrated associations between imaging and gene expression (14). These insights have been used to predict response to treatment of gliomas (9). Further, by forming clustering patterns on structural MRI across patients, these patterns can be used to identify GBM phenotypic subtypes (15). In addition to the molecular and genetic features, the synaptic input of neurons on glioblastoma cells has been shown to be a powerful influence of promoting tumor growth (16). Currently, there is no imaging biomarker of this synaptic interaction.

Increasingly, it has become clear that brain networks and their alterations associated with GBM have an impact on survival. Stoecklein et al. demonstrated that resting-state functional connectivity (rsFC) measured by MRI is affected by gliomas throughout the whole brain and this information indicated individual glioma disease burden (17). Daniel et al. took these findings further to show that functionally connected voxels can be routinely found within glioblastoma tumors and that intra-tumor connectivity strength is a prognostic marker for overall survival (18). What is currently lacking is a methodology that leverages these group-level scientific findings and provides an actionable imaging biomarker that can inform and guide clinical care of individual patients.

In this study, we examined whether rsFC between pre-defined regions of interest (ROIs) can enable machine learning algorithms to predict overall survival (OS) for individual patients. For this, we used resting-state functional MRI with blood oxygen level-dependent (BOLD) signals acquired in 64 de novo GBM patients prior to standard of care treatment with surgery and chemoradiation. Retrospectively, patients' clinical course and rsFC between ROIs trained a support vector machine (SVM) to predict OS. In this work we provide evidence that the alterations of functional organization of the brain can provide insights into predicting a GBM patient's oncologic course.

## Materials and Methods

### Participants

A total of 64 patients with a pathologic diagnosis of GBM were included in this study. Patients were recruited from the neurosurgery brain tumor service, initially as part of a National Institutes of Health (NIH) funded tumor database project (NIH 5R01NS066905). Inclusion criteria stipulated that each patient was newly diagnosed with a brain tumor, that they underwent surgical treatment of the tumor, that the pathology was GBM, and that there was a pre-surgical indication for structural MRI and resting-state functional MRI as determined by the treating neurosurgeon. Exclusion criteria were age <18 years and prior surgery for a brain tumor. All patients provided written informed consent and the study was approved by the Institutional Review Board of the Washington University in St. Louis.

#### Group Construction and Patient Demographics

GBM patients were classified into two groups with respect to the median duration of OS: 14.6 months (1). Patients surviving <14.6 months were grouped as short-term survival (STS) while those surviving 14.6 months or longer were grouped as long-term survival (LTS). Demographic, clinical and molecular characteristics of STS and LTS groups are summarized in Table 1.

**Table 1:** Patients' demographic, clinical, and molecular characteristics of patient population by group.

**Summary of characteristics**			
	**Short term survival (*****n*** **=** **35)**	**Long term survival (*****n*** **=** **29)**	***P*****-value**
Age in years (range)	62.6 ± 11.6 (27–83)	58.5 ± 9.1 (41–79)	0.114
**Sex**			
Male	21 (60.0%)	24 (82.8%)	0.058
Female	14 (40.0%)	5 (17.2%)	
CE volume (cm^3^)	37.6 ± 28.7	22.9 ± 28.7	0.004
FLAIR volume (cm^3^)	109.4 ± 67.6	83.4 ± 76.5	0.074
**KPS**, ***n*** **(%)**			
>70%	6 (21.4%)	13 (52.0%)	0.025
Missing	7	4	
**Extent of resection**			
Gross-total	11 (31.4%)	10 (34.5%)	
Subtotal	13 (37.1%)	14 (48.3%)	
Laser	7 (20.0%)	1 (3.5%)	
Biopsy	4 (11.4%)	4 (13.8%)	
**MGMT status**			
Methylated	12 (37.5%)	13 (52.0%)	0.297
Non-methylated	20 (62.5%)	12 (48.0%)	
Missing	3	4	
**IDH mutation**			
Mutated	0	0	
Wild type	34	29	
Missing	1	0	
**EGFR amplification**			
Positive	6 (30.0%)	12 (63.2%)	0.056
Negative	14 (70.0%)	7 (36.8%)	
Missing	15	10	
Overall survival in days	242.1 ± 118.0	840.9 ± 372.6	<0.00001

*STS, short-term survival (<14.6 months); LTS, long-term survival (≥14.6 months); CE, contrast enhancement; FLAIR, fluid-attenuated inversion recovery; KPS, Karnofsky performance status; MGMT, methylguanine methyltransferase; IDH, isocitrate dehydrogenase; EGFR, epidermal growth factor receptor*.

### MRI Acquisition

Imaging was done on Siemens Trio or Skyra MRI scanners operating at 3T. Patients were scanned using a standard presurgical tumor protocol. Structural imaging included T1-weighted magnetization prepared rapid acquisition gradient echo (MPRAGE) and T2-weighted fast spin-echo. Resting-state functional MRI was acquired using echo-planar imaging sequences (voxel size = 3 mm cubic; echo time = 27 ms; repetition time = 2.2–2.9 s; field of view = 256 mm; flip angle = 90°) for a total of 320 frames.

### Resting-State Functional Connectivity (rsFC) Pre-processing

We used standard pre-processing approaches previously described (19, 20). Denoising methods included slice timing corrections which removed systematic slice intensity differences due to interleaved acquisition, and head motion corrections within and across runs. Atlas transformations were achieved by the composition of affine transforms connecting functional imaging volumes with T2-weighted and T1-weighted structural images. Thereby, we registered volumetric BOLD time series to an isotropic 3 mm atlas space. Additional preprocessing included spatial smoothing (isotropic 6 mm full-width half-maximum Gaussian blur), removal of linear temporal trends from voxels in each scanning run, and temporal low-pass filtering to retain frequencies <0.1 Hz. Spurious variances were reduced by regression of nuisance waveforms derived from head motion correction and time series sampled from regions of white matter and cerebrospinal fluid. The whole-brain (global) signal was removed as a nuisance regressor. Frame censoring was performed to minimize the impact of head motion on FC computations. Thus, for each volumetric frame, if the root-mean-square of voxel intensities within brain regions changed significantly compared to the previous frame, the frame was censored. Significant changes were defined as those that exceeded 0.5% of root-mean-square voxel intensities.

### Resting-State Functional Connectivity (rsFC) Analysis

The BOLD time series for pre-defined volumetric ROIs were obtained by averaging the voxel time series within each ROI. The rsFC between any pair of ROIs was then defined as Pearson's product moment correlation coefficient between ROI-specified time series. We used the set of 300 ROIs from the study by Seitzman et al. [see (21), for detail]. Briefly summarized, this set of 300 spherical ROIs comprise 264 previously described ROIs (22) with the addition of subcortical and cerebellar ROIs. Thus, the ROI set comprised 239 cortical, 34 subcortical, and 27 cerebellar ROIs. The cortical ROIs belong to one of 13 resting-state networks (RSNs): the cingulo-opercular network (CO), frontoparietal network (FPN), dorsal attention network (DAN), ventral attention network (VAN), salience (SAL) network, somatomotor dorsal (SMD) network, somatomotor lateral (SML) network, visual (VIS) network, auditory (AUD) network, medial temporal lobe (MTL) network, reward (REW) network, parietomedial (PMe) network, the default-mode network (DMN), and cerebellum regions (all cerebellum ROIs). Finally, we identified a collection of twelve ROIs which overlapped with atlas regions for white matter and tentorium, excluding their assignment to any of the 13-resting state functional network. This was consistent with methodological precedence for avoiding confounding of resting-state inferences by ROIs encompassing non-gray matter (22), also see Supplementary Material. Thus, for each subject, a 288 × 288 functional connectivity (FC) matrix was computed.

### Classification Using Machine Learning

**Figure 1:**
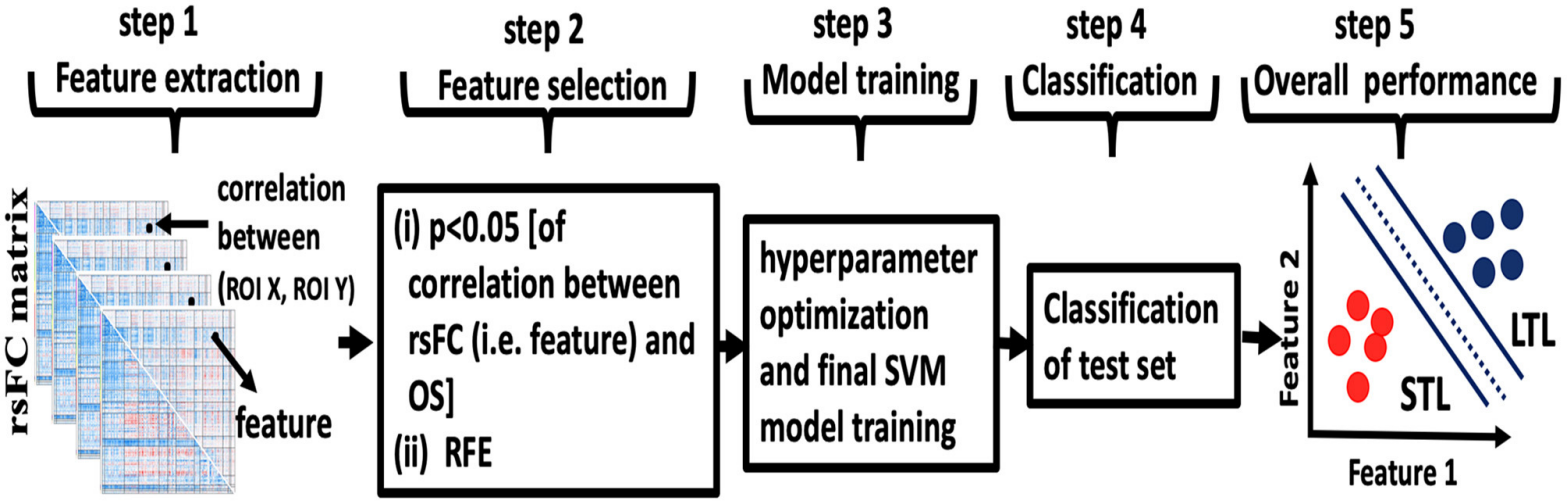
Overview of machine learning. **Step 1**: the rsFC matrix of size 288 × 288 was computed for each patient. The features were rsFC between pairs of ROIs (for example, the correlation between ROI X and ROI Y represented by dots in step 1). **Step 2**: feature selection was performed in two steps using only the lower triangular rsFC matrix to exclude self and symmetric connections. First (i), features with correlation with patients' days of overall survival OS (*p* < 0.05, uncorrected) were selected for heuristic filtering. Then (ii), recursive feature elimination (RFE) was used to select the best predicting feature subset. **Step 3**: hyperparameter optimization and final SVM model training was performed. **Step 4**: the trained model from the cross-validation fold was tested against the single held-out subject. Steps 1–4 were repeated for each fold of leave-one-out cross-validation. **Step 5**: following all cross-validation folds, accuracy, AUC, sensitivity, and specificity for the full sample set were computed.

We used the Caret package available within RStudio to implement our machine learning classifier [(23), rstudio.com]. We used a support vector machine (SVM) with linear kernel because of its favorable reported predictive performance in medical knowledge discovery with small amounts of training data (24). Because of the limited number of patients in the present study and our aim to minimizing bias in the estimate of classification accuracy, we used the leave-one-out (LOO) cross-validation method. An overview is illustrated in Figure 1. Our use of LOO cross-validation adhered to standard prescriptions for cross-validation implemented in the Caret package. For pedagogical overviews of cross-validation and SVM we recommend the cross-disciplinary textbook by Hastie et al. (25).

This analysis included 64 patients, thus 64-folds of LOO cross-validations. For example, for the first cross-validation fold, all data from one patient were withheld and data from the 63 remaining patients were used for feature selection, training the SVM and tuning hyperparameters. Then, the fold-1 model was tested against the held-out data. In the next cross-validation fold, all data from a different patient were withheld and data from the 63 remaining patients were used for feature selection, retraining the SVM and retuning hyperparameters. Then, the fold-2 model was tested against the held-out data and so on until we predicted (tested) all 64-subjects by running such 64-folds.

The Pearson product moment correlation of 288 ROIs specified in section Resting-State Functional Connectivity (rsFC) Analysis is represented by a correlation matrix of size 288 × 288 (Figure 1, step 1). Diagonal matrix elements are exactly unity and non-informative while upper triangular matrix elements are symmetric to lower triangular matrix elements. Consequently, we count the informative correlations over ROIs to be 41,328, equivalent to number of combinations $\left(\frac{288}{2}\right)$. We defined the Fisher z-transformation of this set of informative correlations to be the original features for rsFC. Since the number of original features, 41,328, is much higher than the number of patients, we used feature selection to avoid overfitting our SVM.

Feature selection was performed in two steps (26, 27) (Figure 1, step 2). First, using all patient training data (except the withheld subject's data), we computed the correlation of original features for rsFC to OS expressed as continuous time of survival. We discarded features with *p*-value >0.05 (uncorrected) for heuristic filtering. The *p*-value for correlations was not used for significance testing of any kind. Second, we used recursive feature elimination (RFE) (28) which also ranks features according to their predictive ability. However, features tend to also be correlated with one another and so the multivariate discriminant classifier retains high dimensionality. RFE repeatedly, and recursively, evaluates the rank of features for predictively ability, eliminating the worst performing features. Within the RFE framework, we used 4-fold internal cross-validation (not to be confused with external LOO cross-validation) with ten iterations to obtain ranked selected features for rsFC. Internal cross-validations and iterations are necessary for RFE to correctly eliminate large numbers of features. Using heuristic *p*-value filtering and RFE, we reduced rsFC features from 41,328 to the best performing features subset. Our strategy submitted these best performing features to SVM.

As with other machine learning algorithms, SVM may perform poorly until hyperparameters are tuned for the problem domain. The caret package enabled tuning SVM scaling and model complexity cost using grid search (Figure 1, step3). Thus, the main parameters, the cost in case of linear SVM, was estimated by using the grid-search algorithm at the scale of *c* = 1:10. We used 4-fold cross-validation for hyperparameter tuning following feature extraction, feature selection (*p*-value filtration and RFE), and in model training. Following training on 63 patients of the training set from LOO, we tested classification performance on the single held-out patient of the test set from LOO (Figure 1, step 4). Upon completion of 64 LOO folds, we ascertained the performance of the final classifier, computing accuracy, specificity and sensitivity values using the standard confusion matrix (see Supplementary Material for detail). To evaluate overall model performance, we also performed Receiver-operating Characteristic (ROC) curve analysis.

## Results

**Figure 2:**
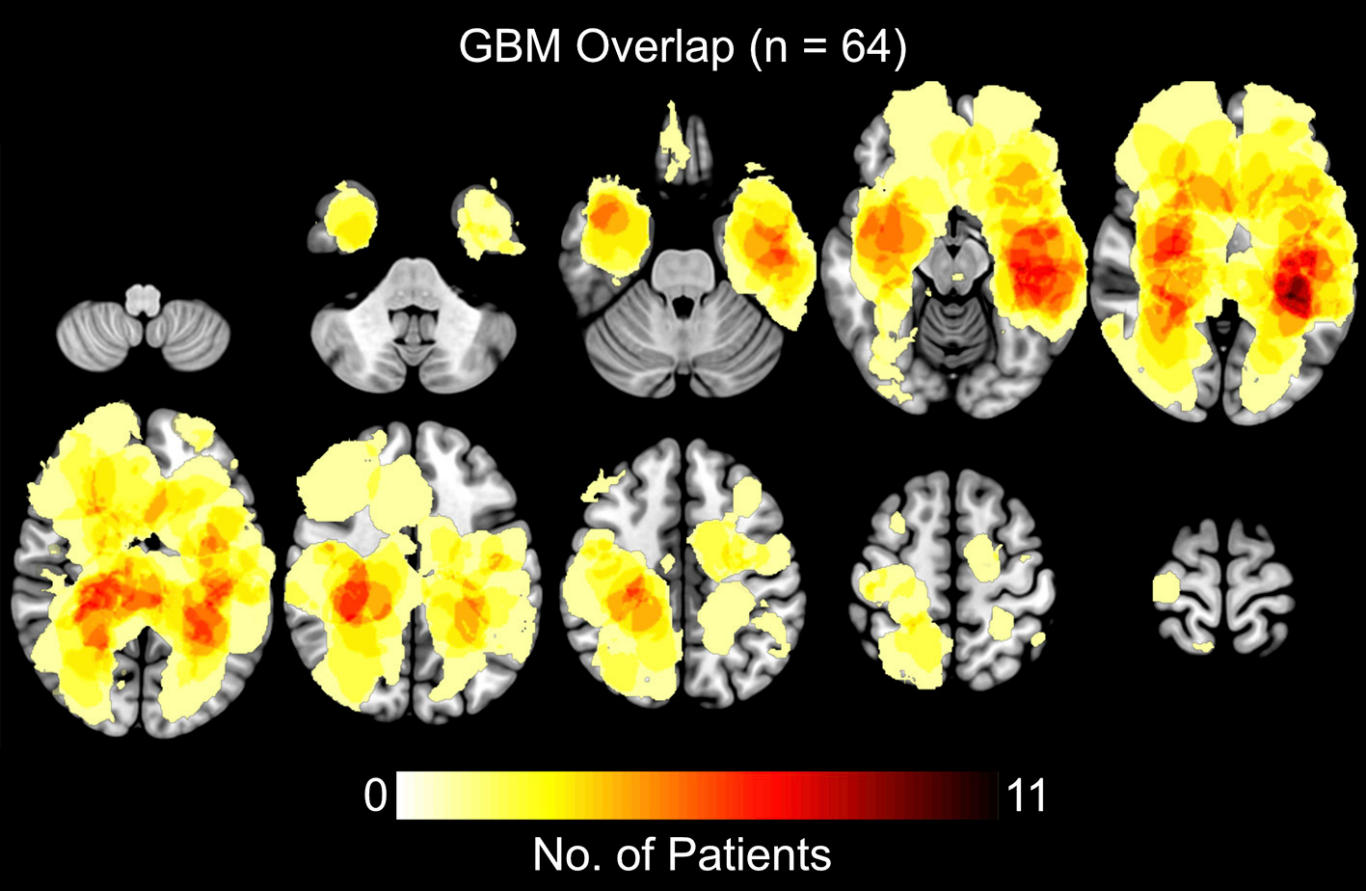
Heatmaps showing the distribution of tumor density, defined by contrast-enhanced (CE) T1w boundaries, in the full sample of 64 patients.

A total of 64 patients diagnosed with *de novo* GBM were partitioned into two groups, STS and LTS, based on overall survival (OS). The summary of patient's clinical, molecular, and genetic characteristic of present sample were also reported in Table 1. To test the significance of differences in summary characteristics between two groups, we performed the Mann-Whitney *U*-test (for the continuous variables; age, CE and FLAIR volume, KPS) and Fisher's exact test (for the categorical variables: Sex, MGMT status, IDH mutation, EGFR) and *p*-value of resultant test were also reported (Table 1). Furthermore, the heterogeneity of GBM location, size, and morphology is illustrated in Figure 2. These heatmaps represent the distribution of tumor density in the entire patient cohort as defined by contrast-enhanced (CE) T1w boundaries segmented by using the software application ITK-SNAP (29). The distribution shows no systematic asymmetry or focality that could significantly bias the results.

### Correlation Between Resting-State Functional Connectivity and Overall Survival (OS)

**Figure 3:**
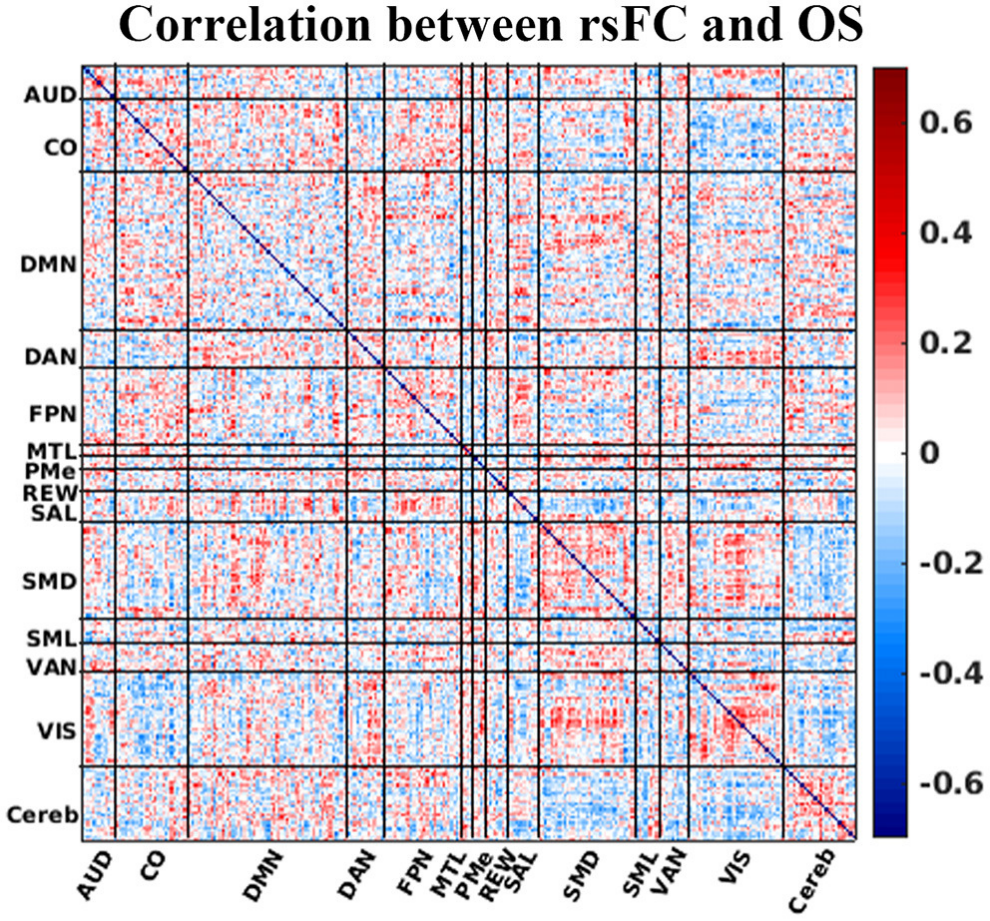
Correlation between rsFC (Fisher's z transformed pairwise ROI correlation) and OS. The horizontal and vertical axis of the plot is the ROI number sorted by the resting-state network. The surface color represents the correlation between OS and ROI-to-ROI rsFC. Correlation strength between ROI pair and OS is represented by colorbar. AUD, auditory; CO, cingulo-opercular; DMN, default-mode network; DAN, dorsal attention network; FPN, frontoparietal network; MTL, medial temporal lobe; PMe, parietomedial network; REW, reward network; SAL, salience network; SMD, somatomotor dorsal network; SML, somatomotor lateral network; VAN, ventral attention network; VIS, visual network; and Cereb, cerebellum regions (all).

Figure 3 illustrates how each element of the matrix of canonical functional connectivities correlates with OS. That is, for each element of functional connectivity between ROIs, the vector of measured functional connectivities for 64 patients was correlated with the vector of days of OS for the patients. Please note that the surface color in the Figure 3 represents the correlation between OS and ROI-to-ROI rsFC. Figure 3 demonstrates that there are no obvious patterns by which OS may be inferred directly from rsFC. Matrix elements in red denoting positive correlations with OS intersperse with matrix elements in blue denoting negative correlations with OS. This absence of semantic patterns motivates techniques of feature reduction and inference by machine learning. The strategy of heuristic filtering using uncorrected *p*-values <0.05 pruned 41,328 unique matrix elements to ~1,550 selected matrix elements (the number may change slightly from the fold to fold of LOO cross-validation). The strategy of wrapping using RFE pruned selected matrix elements to just 60 using a principled feature reduction technique (28). The 60 final matrix elements were given to SVM for classification (also see Supplementary Material for details)

### Machine Learning Classification of Short-Term Survival (STS) and Long-Term Survival (LTS) GBM Patients

**Figure 4:**
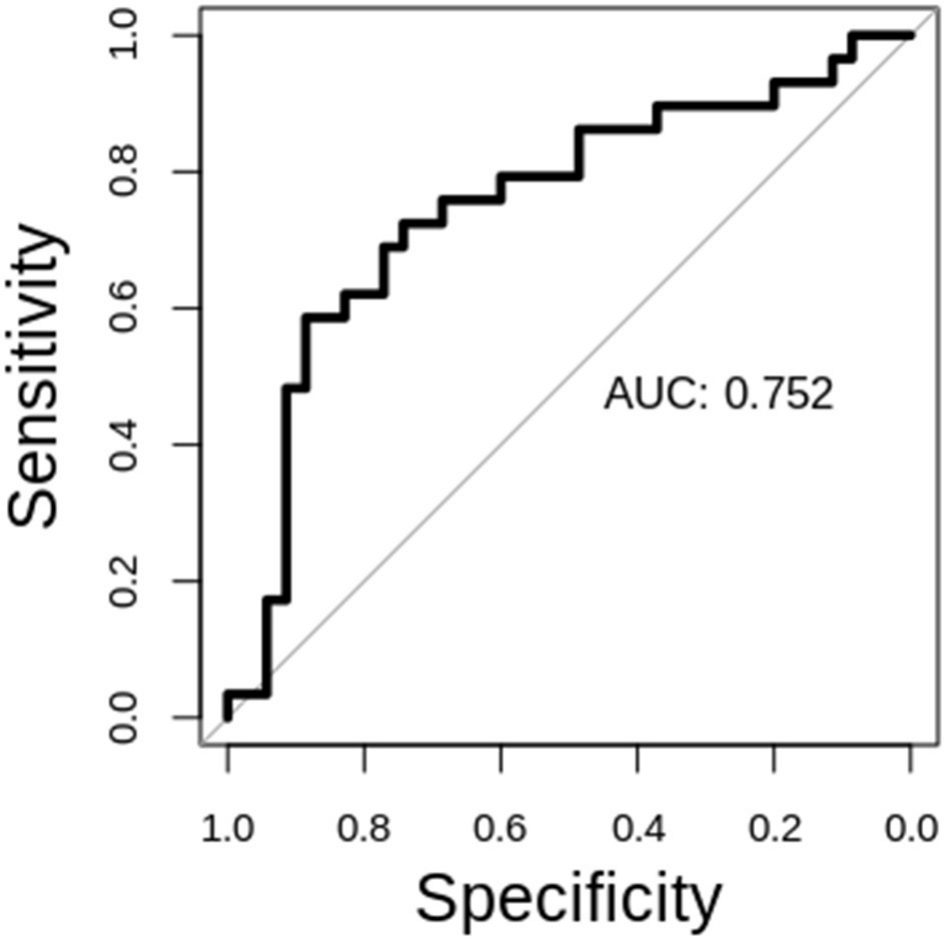
ROC analysis for the STS vs. LTS classification.

The performance of our classification schemes in predicting short vs. long term survival are presented in Table 2. Briefly; within-patient classification accuracy was 71.88%. Similarly, the sensitivity and specificity were 77.14 and 65.52%, respectively. The area under the curve (AUC) value was 0.752 (95% CI, 0.62–0.88). The receiver operating characteristic (ROC) curves for stratifying patients is shown in Figure 4.

**Table 2:** Classification performance summary of SVM classifier.

**SVM (LOO)**	**Accuracy**	**Sensitivity**	**Specificity**	**AUC**	***C***
STS vs. LTS	71.88%	77.14%	65.52%	0.752	1

*Regarding C, although we used the grid-search algorithm at the scale of c = 1:10 to optimize C, all selected C were 1*.

### The Most Predictive Features

**Figure 5:**
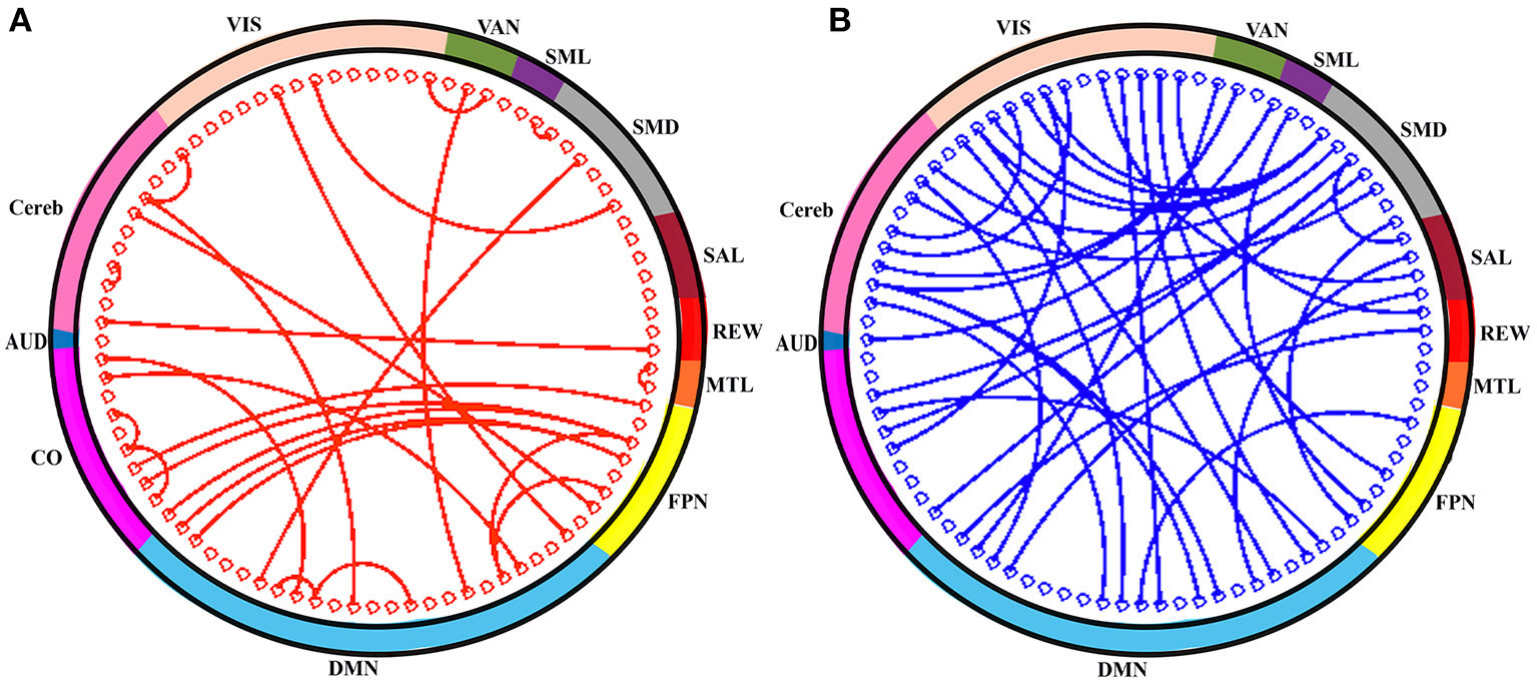
The top-60 frequently selected features are shown in circle. **(A)** Red connection (line) indicates the positive correlation of connections (i.e., rsFC) with OS and that of negatively correlated connections are in blue **(B)**. The color of the outer sphere represents the resting-state network (RSN) that the ROIs belongs to. AUD, auditory; CO, cingulo-opercular network; DMN, default-mode network; FPN, frontoparietal network; MTL, medial temporal lobe; REW, reward network; SAL, salience network; SMD, somatomotor dorsal network; SML, somatomotor lateral network; VAN, ventral attention network; VIS, visual network; and Cereb, cerebellum regions (all).

In order to assess the predictive contribution of each features, we sorted the top 60 contributing features as follows. During model training, feature selection was performed on the training set within each LOO fold, producing variations of selected features across cross-validation folds. We stored the 60 top-ranking features from SVM-RFE from each LOO fold. We then sorted features according to their frequency of occurrence in all 64 LOO folds. Figure 5 illustrates, as network adjacency maps, the RSN distribution of the selected features using vertices for ROIs (node) and edges for correlations of selected features with OS. Of the top 60 features, 27 were reproducibly found in all 64 LOO folds, invariant to tumor and physiologic variability across patients [for completeness, anatomical distribution of these top 60 and 27 shared features (separately) were also plotted, see Supplementary Figures 1, 2, respectively].

## Discussion

Anatomic and functional imaging currently is routinely utilized prior to and during the resection of brain tumors. This technology has been shown to improve the extent of tumor resection (30), and as a result, improve survival statistics (31). That said, it is not routine prior to resections to make use of imaging that reflect the functional organization of the brain and its interaction with the tumor to provide insight into long-term prognosis. Beyond, guiding the specific surgery *per se*, deep insight into the aggressiveness of the tumor informs fundamental decision making about the total course of care. Historically, task-based fMRI has been employed as a means of pre-operatively localizing function (32). During the past decade, it has been shown that the representation of multiple motor, sensory, and cognitive functions can be mapped by analysis of intrinsic brain activity, acquisition of which requires only that the patient hold still during fMRI (33–35). Thus, resting-state fMRI provides a much more complete functional map of the brain than does task-based fMRI; moreover, rs-fMRI is more reliable and much more time-efficient. Finally, the robustness of this mapping modality enables the identification of functional tissue both within a tumor (providing insight into glial-neuronal interactions) and throughout the brain (providing measures of global functional distortions (17, 18, 36–38), What has remained a challenge has been dealing with the complexity and magnitude of the resting-state fMRI data to provide reliable and actionable insights that can enhance clinical care.

Machine learning approaches creates the opportunity to organize large amounts of data to support more generalized and actionable interpretations. In the context of prognostic radiomics for GBM there are several considerations that merit attention. First, in the supervised context, as done in this work, predictions were formulated by direct comparisons of abundant functional data and outcome measurements. Recursive feature elimination (RFE) with support vector machines (SVM) were chosen because they outperformed many popular classification algorithms in a survey of neuroimaging studies of brain disorders: simple thresholding, centroid methods, minimum distance, discriminant function analysis, Gaussian process, spectral clustering, fused lasso, random forests, perceptrons, stacked auto-encoder neural networks, SVM without RFE (39, 40). Second, appropriately selecting features, which determine the dimensionality of a machine learning model, is critical for SVM in the face of limited outcome data, such as the OS of GBM patients. Valid selection of features can help increase prediction accuracies and can also help interpretability. This work made use of a heuristic filtering method that calculated correlations and used uncorrected *p*-values to prune features. RFE then served as a wrapper method to further prune features that were most appropriate for the problem of predicting OS for GBM patients. Third, a linear SVM provided classification. While our feature selection choices provided limited interpretability of feature subsets (network topographies and patterns in neuroanatomy were not evident), feature selection did improve clinically relevant prediction accuracies. Specifically, this work demonstrated that patients with GBM can be partitioned into short term and long-term survival groups using features extracted from resting-state fMRI (Table 2 and Figure 4). These findings complement previous work demonstrating the potential of rsFC as a biomarker of OS in GBM patients (18). Notably, this work demonstrates predictions of OS for individual patients.

While the prediction of OS is clinically important a caution is warranted in interpreting the features used to make those predictions because of the nature of support vectors in determining decision hypersurfaces in high-dimensional data. The 60 most frequently selected features are plotted in Figure 5 (anatomical distribution of these features is also plotted, see Supplementary Figure 1). Unlike other methods, such as linear discriminant analysis in which features are ascribed shared covariances that occupy the space of dominant features, our use of RFE-SVM selects features which serve as kernel bases optimally separating features which are most ambiguous along the decision boundary. Consequently, while having optimal benefits for generating decision hypersurfaces, our selected features for rsFC in fact visualize anatomy and network adjacencies that are most ambiguous for predicting OS. The difficulty of interpreting features selected by machine learning algorithms is a common problem of neuroimaging research. Many features contribute to the classification of OS because of the complexity of factors that determine OS. Broadly distributed features have been found in many previous investigations of rsFC in GBM patients (41–43).

FC studies have markedly advanced our knowledge of human brain function and its organization. Thus, rsFC has been used to characterize individuals' functional brain organization in patients with a broad range of neurosurgical diseases including GBM. Relatively recently, there were reports of significant alteration of brain functional connectivity in GBM (42, 43). We, for the first time, directly tested the relationship of such changes with OS in GBM patients (Figure 3). We emphasize the advantages of rsFC over task-based MRI. For instance, resting-state functional MRI can be acquired in patients that are unable to cooperate with a task, such as cognitively impaired patients as they do not need to perform a task (20, 44, 45). Moreover, task-based fMRI conventionally is restricted to mapping the representation of motor and speech function, which omits other important functions (e.g., executive function, attention, etc.) and does not perform a whole brain assessment. Even the waking state during fMRI is not required as essentially the same functional maps are obtained even if the patient is asleep or sedated (46–48). Thus, rsFC provides information complementary to that obtained from structural imaging of brain tumors. Notably, rsFC throughout the brain is affected by gliomas, even in the non-lesional hemisphere (41–43). This was one of the key motivations for this study.

To our knowledge, no previous studies have examined correlations between rsFC and individual OS as a filtering heuristic. While weak correlations have no role for testing hypothesis, they can provide intermediate data for consumption by more principled algorithms, such as RFE and SVM. These weak correlative relationships may be interpretable in the context that functional connectivity across networks correlates with a patient's cognitive function (41, 49, 50). In cases of high grade GBM, reports describe marked decline in neurocognitive functioning during the course of a patient's disease (51). Moreover, poorer performance on initial cognitive testing is associated with shorter survival (52). Here we demonstrate that a trained SVM predicts short and long-term survival in GBM patients based on rsFC measures. Previous work in GBM patients have shown that cognitive impairment in GBM patients can be associated with both increases and decreases in rsFC (53, 54).

The ability of several rsFC ROI pairs to predict OS further extends our previous report that intratumor rsFC may be a prognostic marker for overall survival (18). This finding aligned with previous reports that brain tumors can lead to various cognitive deficits and are related to alterations in local and interhemispheric rsFC across functional networks (49, 50, 55, 56). Here we showed that particular ROI-pair dependent reductions and increases in rsFC are associated with OS prediction, suggesting that connectivity alterations at specific cortical locations play an important role in influencing outcomes. Thus, local and global rsFC changes in GBM patients may act as a biomarker for prognosis and disease monitoring.

## Limitations

A common deficiency of neuroimaging studies is limitations of sample size in the presence of data with high dimensional sets of features (57). The difficulties of machine learning methods under these restrictions include overfitting and the inability to adequately represent the complexity of the underlying study problem. Feature selection did not account for tumor location, tumor staging, tumor grading, and aspects of patient demography, such as age, sex, and ethnicity. However, inclusion of such additional features in the classifier are likely to improve the performance of the classifier described in this work. There remain issues regarding reproducibility and generalizability of results. Partial solutions include recruiting larger numbers of patients for study and testing models with unseen data. In this work, we used LOO on all available patient data.

## Conclusion and Future Work

In summary, our results demonstrate that resting-state functional connectivity provides prognostic biomarkers for individual patients with GBM. This work demonstrates prognostic classification of short-term survival vs. long-term survival and suggests how future work may attain more precise predictions of years of survival for individual patients. Such efforts may require extensive longitudinal data to attain clinical utility but such precision predictions would have substantial and meaningful impact for patients with GBM making decisions for their terminal care.

## Data Availability Statement

Tumor data will be made available upon request to Eric C. Leuthardt.

## Ethics Statement

The studies involving human participants were reviewed and approved by Institutional Review Board of the Washington University in St. Louis. The patients/participants provided their written informed consent to participate in this study.

## Author Contributions

BL and EL designed the study. AD, BL, and DM collected and assembled the data. JL and JS assisted in pre-processing of the data. BL and AD performed the post-processing data analysis. BL prepared the first draft. All authors critically reviewed and edited the manuscript.

## Conflict of Interest

EL has equity in Neurolutions, Inner Cosmos, and Sora Neuroscience. Washington University has equity in Neurolutions. DM owns stock in Radiologics and Sora Neuroscience. The remaining authors declare that the research was conducted in the absence of any commercial or financial relationships that could be construed as a potential conflict of interest.
