# Supplementary material for: Machine Learning Analytics of Resting-State Functional Connectivity Predicts Survival Outcomes of Glioblastoma Multiforme Patients
Source: Front Neurol. 2021 Feb 22;12:642241. doi: 10.3389/fneur.2021.642241 (PMC7937731; doi:10.3389/fneur.2021.642241)
Supplement: Supplementary file 1 [file Data_Sheet_1.DOCX]

**Machine Learning Analytics of Resting-State Functional Connectivity**

**Predicts Survival Outcomes of Glioblastoma Multiforme** **Patients**

Bidhan Lamichhane*^1^, Andy G. S. Daniel^2^, John J. Lee^3^, Daniel S. Marcus^3^, Joshua S. Shimony^3^ and Eric C. Leuthardt *^1,2,4,5,6,7^

**Affiliations:**

1 Department of Neurological Surgery, Washington University School of Medicine, St. Louis, MO 63110, USA

2 Department of Biomedical Engineering, Washington University in Saint Louis, St. Louis, MO 63130, USA.

^3^Mallinckrodt Institute of Radiology, Washington University School of Medicine, St. Louis, MO, United States

4 Department of Neuroscience, Washington University School of Medicine, St. Louis, MO 63110, USA.

5 Department of Mechanical Engineering and Materials Science, Washington University, St. Louis, MO 63130, USA.

6Center for Innovation in Neuroscience and Technology, Washington University School of Medicine, St. Louis, MO 63110, USA.

7 Brain Laser Center, Washington University School of Medicine, St. Louis, MO 63110, USA

***Corresponding authors**: Eric C. Leuthardt, [leuthardte@wustl.edu](mailto:leuthardte@wustl.edu)

Bidhan Lamichhane, [bidhanlamichhane@wustl.edu](mailto:bidhanlamichhane@wustl.edu)

**Keywords**: Brain tumor, resting-state functional connectivity, biomarker, overall survival, short and long-term survival, classification, support vector machine

**Feature selection**

As we used leave-one-out (LOO) cross-validation approach, the outer-most loop of LOO selected 63 patients for training and one patient for testing per LOO fold. An inner loop, RFE was used to rank and reduce the features into the best performing subset following the first step of feature reduction by correlation analysis. We evaluated 40 to 100 top-ranked subset features with a stride of 10 features. That is, subsets consisted of the top-ranked 40,50,60,70,80,90, and 100 features. Following LOO, the area under the receiver operating characteristic curve (AUC) quantified the classifier's performance. Table S1 summarizes the outcomes of exploratory features analyses. The best performing subset, which comprised 60 top features, was retained in the final analysis and reported in the main text.

**Table S1:** The performance of SVM at various no of maximum features.

**The confusion matrix**

|  | Actual Positives | Actual Negatives |
| --- | --- | --- |
| Positive Prediction | True Positives (TP) | False Positives (FP) |
| Negative Prediction | False Negatives (FN) | True Negatives (TN) |

**Table S1:** The confusion matrix.

The accuracy describes the ratio of the number of correctly classified patients to the total sample of patients: (TP+TN)/(TP+TN+FP+FN). The sensitivity, or true positive rate, is TP/(TP+FN). The specificity, or true negative rate, is TN/(TN+FP).

**The most predictive features**

**
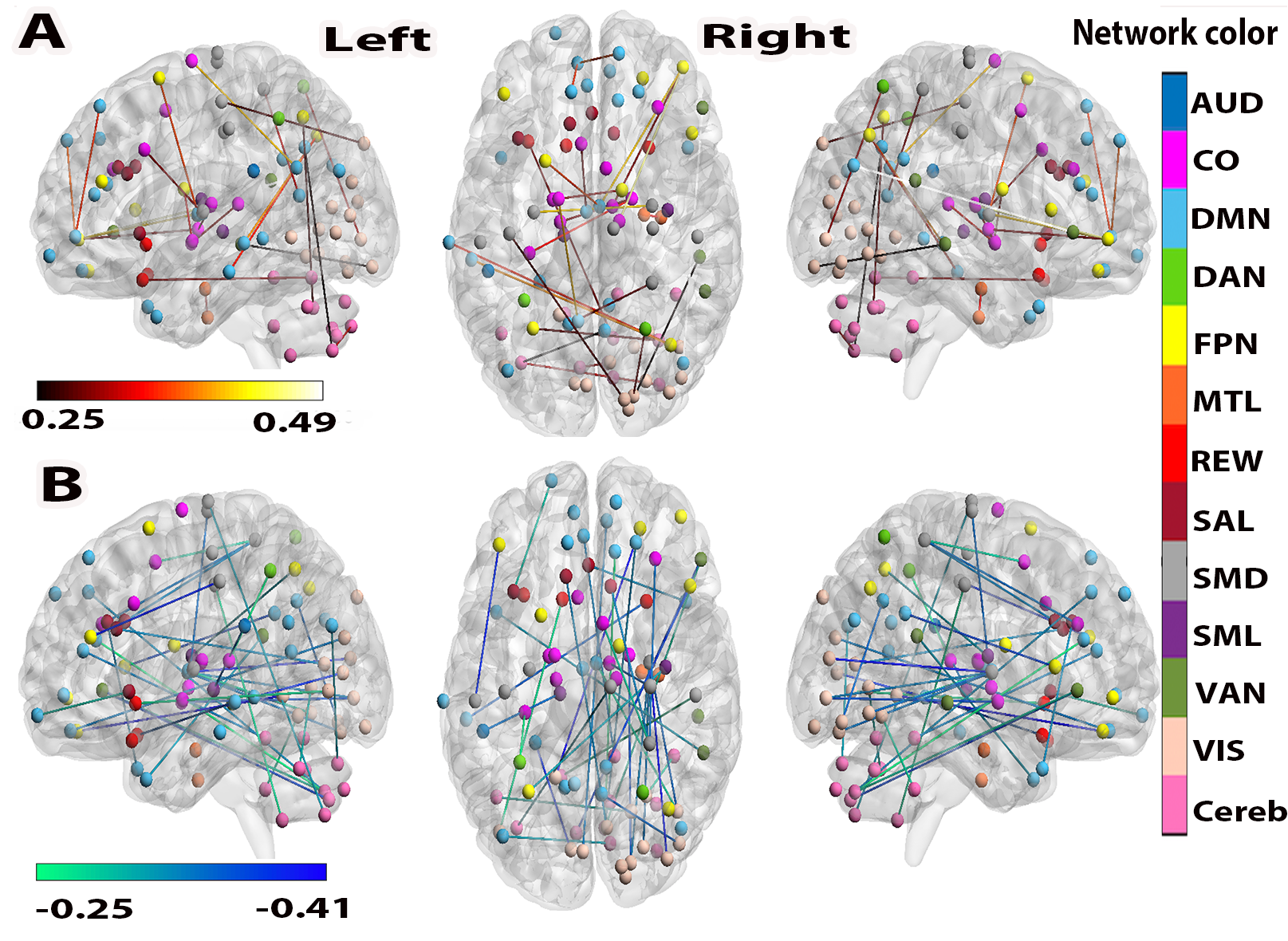
**

**Figure S1.** The top-60 frequently selected features. (A) The features which are positively correlated with OS and that are negatively correlated with OS (B). The line (edge) color represents the correlation of rsFC between nodes and overall survival (OS). The color of nodes (sphere) represents the resting-state network (RSN) that the node belongs to. The BrainNet Viewer package (Xia et al., 2013), available at <http://www.nitrc.org/projects/bnv/>, was used to map ROIs (node) and rsFC(edge) onto the cortical surfaces. Please note that nodes that were not connected to other nodes are also a part of the 60 selected features. Thus, each node has at least one connection either in Figure S1A or in Figure S1B.

**27 features that were found in all 64 LOO folds:**

**
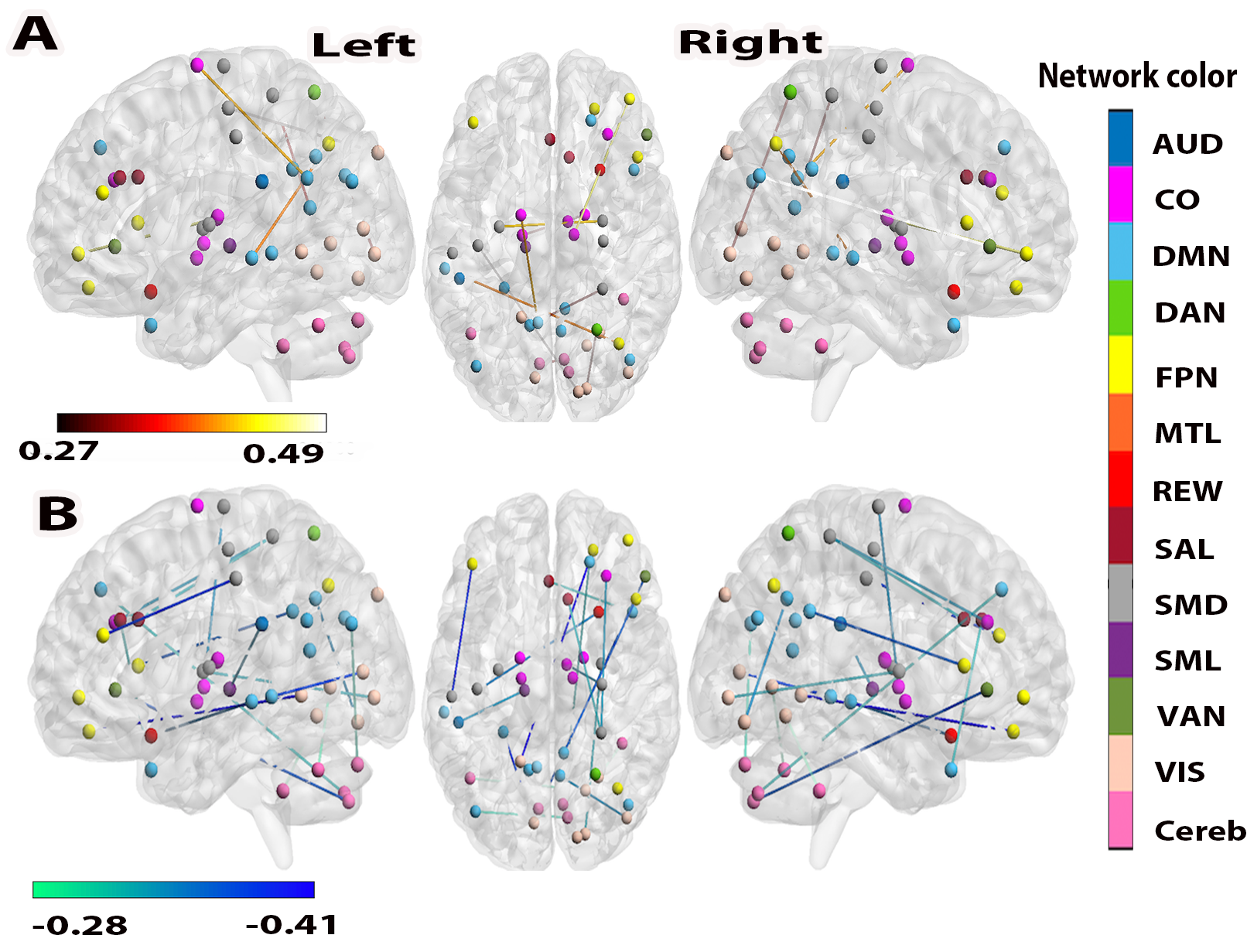
**

**Figure S2.** The 27 features that were reproducibly found in all 64 LOO folds (A) The features which are positively correlated with OS and that are negatively correlated with OS (B). The line (edge) color represents the correlation of rsFC between nodes and overall survival (OS). The color of nodes (sphere) represents the resting-state network (RSN) that the node belongs to. Please note that nodes that were not connected to other nodes are also a part of the 27 features. Thus, each node has at least one connection either in Figure S2A or in Figure S2B.

**12-unassigned ROIs (out of 300 sets)**

As mentioned in the main text, we excluded twelve regions of interest in this analysis. Mainly because they overlapped with atlas regions for white matter and tentorium and were not included in the 13-resting state functional networks. The MNI cordinates of 12-unassigned ROIs were reported on the Table S2.

For detail, see Power et al.,(Power et al., 2011) and Seitzman et al., (Seitzman et al., 2020). Also, at <https://wustl.app.box.com/s/twpyb1pflj6vrlxgh3rohyqanxbdpelw>.

| ROI no | MNI coordinates [X,Y,Z] |
| --- | --- |
| 1 | 56.16 , -44.76 , -24.23 |
| 2 | -24.66, -97.84 , -12.33 |
| 3 | 8.13, 41.12 , -24.31 |
| 4 | 26.68 , -97.3 , -13.49 |
| 5 | 48.52 , -2.85, -38.49 |
| 6 | 51.79, -34.17, -27.23 |
| 7 | -57.97, -25.69 , -14.73 |
| 8 | 55.18 , -30.8, -16.93 |
| 9 | 64.6 , -24.41 , -18.57 |
| 10 | -50.06, -7.09 , -39.24 |
| 11 | 33.55 , 38.46 , -12.03 |
| 12 | -46.68 , -50.91 , -20.91 |

Table S2: MNI coordinates of 12-unassigned ROIs (out of 300 set of ROIs).

**Reference**

Power, J. D., Cohen, A. L., Nelson, S. M., Wig, G. S., Barnes, K. A., Church, J. A., Vogel, A. C., Laumann, T. O., Miezin, F. M., Schlaggar, B. L., & Petersen, S. E. (2011). Functional Network Organization of the Human Brain. *Neuron*, *72*(4), 665–678. https://doi.org/10.1016/j.neuron.2011.09.006

Seitzman, B. A., Gratton, C., Marek, S., Raut, R. V., Dosenbach, N. U. F., Schlaggar, B. L., Petersen, S. E., & Greene, D. J. (2020). A set of functionally-defined brain regions with improved representation of the subcortex and cerebellum. *NeuroImage*, *206*(October 2019). https://doi.org/10.1016/j.neuroimage.2019.116290

Xia, M., Wang, J., & He, Y. (2013). BrainNet Viewer: A Network Visualization Tool for Human Brain Connectomics. *PLoS ONE*, *8*(7). https://doi.org/10.1371/journal.pone.0068910
